# *In vitro* interactions between the ectomycorrhizal *Pisolithus tinctorius* and the saprotroph *Hypholoma fasciculare* fungi: morphological aspects and volatile production

**DOI:** 10.1080/21501203.2021.1876778

**Published:** 2021-02-27

**Authors:** Paula Baptista, Paula Guedes de Pinho, Nathalie Moreira, Ricardo Malheiro, Francisca Reis, Jorge Padrão, Rui Tavares, Teresa Lino-Neto

**Affiliations:** a Centro De Investigação De Montanha (CIMO), Instituto Politécnico De Bragança, Campus De Santa Apolónia, Bragança, Portugal; b UCIBIO-REQUIMTE/Laboratory of Toxicology, Faculty of Pharmacy, University of Porto, Porto, Portugal; c BioSystems & Integrative Sciences Institute (Bioisi), Plant Functional Biology Centre, University of Minho, Campus De Gualtar, Braga, Portugal

**Keywords:** Interaction, ectomycorrhizal, saprotroph, fungi, antagonism

## Abstract

Ectomycorrhizal fungi are crucial for forests sustainability. For *Castanea sativa*, ectomycorrhizal fungus *Pisolithus tinctorius* is an important mutualist partner. Saprotrophic fungi *Hypholoma fasciculare*, although used for biocontrol of *Armillaria* root disease, it negatively affected the interaction between the *P. tinctorius* and plant host roots, by compromise the formation of *P. tinctorius-C. sativa* mycorrhizae. In this work, fungal morphology during inhibition of *H. fasciculare* against *P. tinctorius* was elucidated. *P. tinctorius* growth was strongly affected by *H. fasciculare*, which was significantly reduced after six days of co-culture and become even more significant through time. During this period, *P. tinctorius* developed vesicles and calcium oxalate crystals, which were described as mechanisms to stress adaption by fungi. *H. fasciculare* produced different volatile organic compounds in co-cultures over time and differ between single or in dual-species. *H. fasciculare* highly produced sesquiterpenes (namely, α-muurolene) and nitrogen-containing compounds, which are recognised as having antimicrobial activity.

## Introduction

Ectomycorrhizal (ECM) symbiosis is a significant component of several forest ecosystems (Courty et al. 2010). This association results in several benefits for the health and growth of host plants, the most important of which are the exploitation of soil water and solutes (e.g. Plamboeck et al. 2007), the protection of roots against soil-borne pathogens and the improvement of soil nutrient uptake, particularly for elements with a low mobility in the soil (P, N and other micronutrients; reviewed by Courty et al. 2010). The role of certain ECM fungi in organic matter decomposition has been also claimed (reviewed by Lindahl and Tunlid 2015). The control of woody plant litter decomposition rates was traditionally given to saprotrophic microbes, which were recognised for their cellulose, lignin and lignocellulose degrading activities (van der Wal et al. 2013). However, evidences of decomposer capacity/strategy for ECM fungi have revealed that they could also take an active part in organic matter decomposition, sharing some functional parameters with saprotrophs (e.g. Shah et al. 2015). Furthermore, both fungi can interact with each other and/or with host plants in a clearly competitive or mutualistic relation. For example, the simultaneous inoculation of *Castanea sativa* seedlings with the saprotrophic fungus *Hypholoma fasciculare* negatively affected the interaction between the ECM *Pisolithus tinctorius* and plant host roots, due to the failure of this later fungus to compete against the saprotroph (Pereira et al. 2011). The belowground fungal interactions that occur among plant roots and several soil microorganisms are indeed highly dynamic and could considerably affect the overall ecosystem function (Werner et al. 2002).

A complex dialog between soil microbiota and plants has a considerable impact on plant health and productivity. The production of root exudates and other plant/fungi signal molecules, including volatile organic compounds (VOCs), are the “words” of this complex “dialogue” between interactors that can result in synergistic, competitive or antagonistic interactions (Werner et al. 2016). In this context, a broad spectrum of VOCs with diverse ecological functions are described as being produced by fungi, resulting in an inhibitory activity against other fungi or plants or in plant growth effects (Schalchli et al. 2011; Casarrubia et al. 2016; Schenkel et al. 2018). The competitive antagonism between mycorrhizal and saprotrophic fungi has been also recognised for long (Gadgil and Gadgil 1975), but few reports have been dedicated to the interactions occurring between ECM and saprotrophic fungi, although some studies have been focused on *Laccaria laccata* interactions with other soil fungi (Werner et al. 2002; Zadworny et al. 2004, 2007). In contrast, several reports have described the antagonism between ECM fungi and plant-pathogens (reviewed by Itoo and Reshi 2013), as well as saprotrophic towards saprotrophic/pathogenic fungi (Schalchli et al. 2011).

The saprotroph *Hypholoma fasciculare* currently named as *Hypholoma acutum* (Sacc.) E. Horak has been described as a potent antagonistic fungus, able to antagonise several soil-borne microorganisms, such as filamentous fungi (Nicolotti and Varese 1996; Nicolotti et al. 1999; Woods et al. 2005), yeasts (Pereira et al. 2013) or even bacteria (de Boer et al. 2010). Based on its ability of antagonising parasitic fungi, such as *Armillaria* spp., field trials where *H. fasciculare* was used for controlling *Armillaria* root disease have already been established (Chapman et al. 2001; Cox and Scherm 2006). However, the outcome of the complex interaction network occurring belowground could interfere, for example, with the establishment of successful symbiotic relations, such as mycorrhization. Previously, we have detected a competitive interaction between ECM (*P. tinctorius*) and saprotrophic (*H. fasciculare*) fungi, which was able to compromise the formation of *P. tinctorius-Castanea sativa* mycorrhizae (Pereira et al. 2011). In this work, mechanisms of *H. fasciculare* inhibition against *P. tinctorius*, two of the most abundant fungi in Portuguese *Castanea sativa* stands (Baptista et al. 2010, 2015), are elucidated by evaluating morphology and mycelial growth rate, as well as the production of volatile compounds in co-cultures over time.

## Materials and methods

### Fungal cultures

*Hypholoma fasciculare* (Huds.) P. Kumm. strain was obtained from the fungal culture collection of the School of Agriculture, Polytechnic Institute of Bragança, Portugal. This isolate was originally obtained from a *Castanea sativa* orchard at Oleiros – Bragança (Northeast Portugal). Fungal isolation and further molecular identification were performed as previously described in Pereira et al. (2011). *Pisolithus tinctorius* (Pers.) Coker and Couch (isolated 289/Marx) were obtained from the University of Tübingen and maintained in Merlin-Norkans agar medium (MMN) at pH 6.6 [NaCl 0.025 g/L; (NH_4_)_2_HPO_4_ 0.25 g/L; KH_2_PO_4_ 0.50 g/L; FeCl_3_ 0.050 g/L; CaCl_2_ 0.50 g/L; MgSO_4_.7H_2_O 0.15 g/L; thiamine 0.10 g/L; casamino acids 1.0 g/L; malt extract 10 g/L; glucose 10 g/L; agar 20 g/L], at 25°C, in the dark.

## Fungal interaction

Fungi were grown in MMN agar medium, at 25°C in the dark for two weeks, to provide mycelium/spores for the establishment of dual cultures. Hyphal plugs (5 mm of diameter) were removed aseptically from the colony margins and used as inocula for the establishment of dual cultures between *P. tinctorius – H. fasciculare* (*Pt-Hf*), carried out in Petri plates (9 cm diameter), containing 10 ml of MMN agar medium (pH 6.6). The plugs were placed 3 cm apart from each other and incubated in the dark, at 25°C. Controls consisted of MMN plates containing two inocula of the same taxa. i.e., P. *tinctorius* – *P. tinctorius* (*Pt-Pt*), and *H. fasciculare* – *H. fasciculare* (*Hf-Hf*). Six replicates of each combination were performed in two independent experiments. To evaluate the production of volatile compounds, dual cultures *Pt-Hf, Pt-Pt* and *Hf-Hf* were established in 50 ml flasks (Duran Gaines Synth, Bioblock), containing 10 ml of MMN agar medium (pH 6.6), sealed with a polypropylene cap with polytetrafluoroethylene/silicon septum (Duran) to adapt to the solid-phase microextraction (SPME) holder. Three replicates of each combination were evaluated; and flasks only containing MMN agar medium were also included as controls.

## Hyphal interaction evaluation

During 19 days of incubation, the mycelia radial growth towards (internal radius) and away (external radius) the interacting fungus was measured. Macroscopic observation of colonies was performed for over 25 days of interaction in order to identify morphological alterations. Hyphae morphology in the interaction zone was evaluated by scanning electron microscopy (SEM) after 25 days of dual cultures incubation. SEM images were obtained from small pieces of agar collected in the interaction zone and fixed in 2.5% (v/v) glutaraldehyde (grade I, Sigma, St. Louis) in 0.1 M 4-(2-hydroxyethyl)-1-piperazineethanesulfonic acid (HEPES, Sigma, St. Louis) buffer, pH 6.8, for 24 hours. Afterwards, the samples were rinsed three times with HEPES buffer and post fixed in 2% (w/v) osmium tetroxide (Sigma, St. Louis) for 2 h at 4°C. The samples were rinsed with HEPES buffer, dehydrated in an ascending acetone series (10% increases, 30 min each) and dried in hexamethyldisilazan (Merck) for 1 min. Samples were mounted on aluminium stubs and coated with gold using a Fisons Instruments sputter coater SC502. Samples were observed using a scanning electron microscope equipped with energy-dispersive X-ray spectroscopy (Leica Cambridge S360), at 15 keV.

## Volatiles sampling

### Headspace solid-phase microextraction conditions

Guided by the results obtained during the fungal growth experiments, the sampling of volatiles took place after 3, 8 and 14 days of fungal interaction. Extraction of volatiles from the flanks was performed using a divinylbenzene/polydimethylsiloxane (DVB/PDMS) 65 µm fibre. After an incubation at 40°C during 5 min (200 rpm), the fibre was exposed to the headspace during 60 min. The use of stationary phase fibres with 65 µm of thickness and coated with DVB/PDMS have been described as the most successful based on our previous work (Carvalho et al. 2014). While keeping the flask and SPME holder in a horizontal position, the fibre was pulled into the needle sheath, the SPME device was removed from the vial and inserted into the injection port of the GC system for thermal desorption at 220°C. After 2 min, the fibre was removed and conditioned for 10 min at 250°C. The headspace solid-phase microextraction (HS-SPME) analyses were performed in triplicate for each fungal combination. The same procedure was followed for a control sample only containing culture medium.

### Standards

Reference compounds: 3-methyl-1-butanol, 2-methyl-1-butanol, (*E*)-2-nonen-1-ol, phenylethanol, 3-methylbutanal, 2-methylbutanal, benzaldehyde, (*E*)-2-octenal, (*E*)-2-decenal, 3-octanone, α-pinene, limonene, β-pinene, linalool, menthol, cloven, valencene, β-caryophyllene, α-muurolene, 3-chloro-4-methoxybenzaldehyde were purchased from Sigma (St. Louis, MO, USA).

### Gas chromatography-ion trap-mass spectrometry analysis (GC-MS)

HS-SPME analyses were performed using a CP-3800 (Varian) gas chromatograph equipped with a VF-5 ms (30 m × 0.25 mm × 0.25 μm) column (Vairan) and Stabilwax-DA fused-silica (60 m × 0.25 mm × 0.25 μm) column (Restek, USA) to check the identity of some compounds found in the first column. The injector port was heated to 220°C and injections were performed in splitless mode. The carrier gas was helium C-60 (Gasin, Portugal), at a constant flow of 1 ml/min. The oven temperature was set at 40°C for 1 min, then increased at 2°C/min to 220°C, and held for 30 min. All mass spectra were acquired in electron impact (EI) mode. Ionisation was maintained off during the first minute. The ion trap detector was set as follows: the transfer line, manifold, and trap temperatures were 280, 50 and 180°C, respectively. The mass ranged from *m/z* 40 to 350, with a scan rate of 6 scan/s. The emission current was 50 μA, and the electron multiplier was set in relative mode to autotune procedure. The maximum ionisation time was 25,000 μs, with an ionisation storage level of *m/z* 35. Analyses were performed in full-scan mode using Varian Saturn 4000 mass selective detector and Saturn GC-MS workstation software version 6.8.

For metabolite identification, reference standards were used, whenever available. Alternatively, a putative identification was performed using the National Institute of Standards and Technology (NIST 14) database spectral library, and a comparison of the experimental and theoretical Kovats index. For semi-quantification purposes, each sample was injected in triplicate, and the chromatographic peak areas (as count amounts) were determined by a reconstructed full-scan chromatogram using for each compound some specific quantification ions (*m/z*). These corresponded to base ion (*m/z* 100% intensity), molecular ion (M^+^), and another characteristic ion for each molecule. Some peaks that are co-eluted in full-scan mode (resolution value <1) can be integrated with a value of resolution >1. Compounds found in the uninoculated MMN medium (control) were used to subtract any possible compounds from the medium in the dual cultures *Pt-Pt, Hf-Hf* and *Pt-Hf*.

## Data analysis

Data from fungal radial growth and the evolution of each volatile (as peak area/1000) will be indicated as the mean with the respective standard error (SD). Differences between means were analysed by ANOVA using SPSS software, version 21.0 (IBM Corporation, New York, U.S.A.) and averages were compared using Tukey test. Significance was denoted by *p* value less than 0.05.

Multivariate data analysis was applied using principal component analysis (PCA). By using PCA, all data set is decomposed in a reduced number of new variables, denominated principal components (PCs), which are linear and orthogonal combinations of the original data expressed into score vectors and loading vectors. Each PC represents successively the most variability captured from the data, translating the relationship amongst all samples by analysing respective scores distribution, and allowing identify the related variables by the interpretation of the loading vectors obtained. Two different PCA were performed, one using the entire chromatogram (untargeted analysis) and the other using all the chromatographic areas of identified compounds (target analysis). All PCA were performed using Matlab version 7.9 (MathWorks, Natick, MA) with PLS Toolbox version 5.5 (Eigenvector Research Inc., Wenatchee, WA).

In untargeted analysis, all raw data suffered a pre-treatment by peak alignment. Each peak was aligned considering the equivalence of the MS fragmentation patterns. To minimise chromatogram handling, baseline correction and noise removal procedures were discarded. Each aligned chromatogram was normalised dividing each scan signal (intensity expressed as counts) by the sum of intensities of all scans. Peak alignment was absolutely needed to reduce variation in retention times that always occurs across different samples. Then, all data were also mean-centred in order to highlight which volatile compounds suffered higher variations and, consequently, can be the most relevant markers explaining the differences detected over the time. To obtain mean-centred data, the signal of each variable was subtracted from the mean response of that variable across all data.

## Results and discussion

### Pisolithus tinctorius – Hypholoma fasciculare *dual-culture assays*

**Figure 1.**
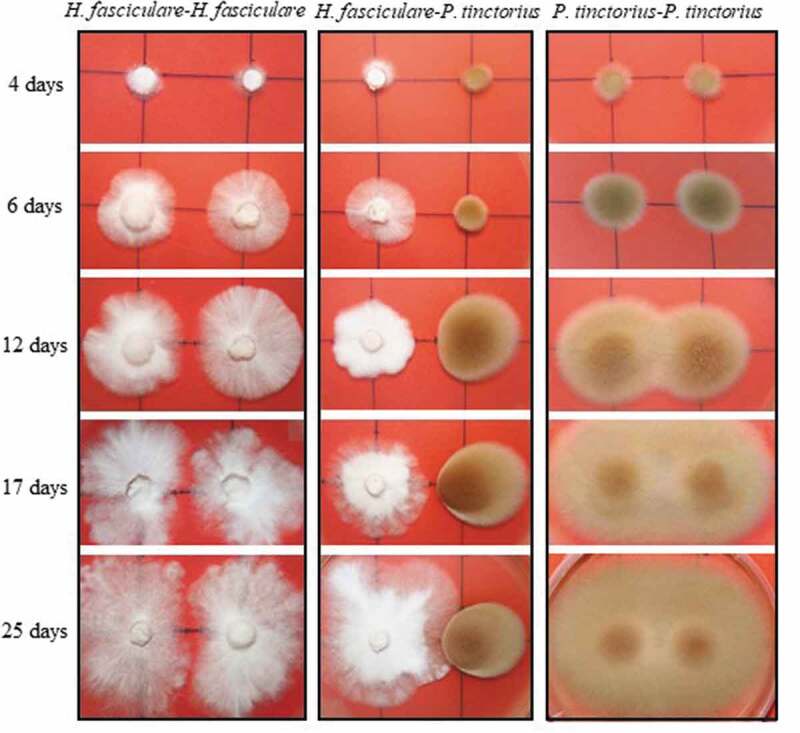
Representative plates depicting the mycelial morphological changes of *P. tinctorius* and *H. fasciculare* during the interspecific interaction (*Pf-Hf* co-culture) and self-paired interactions (*Pt-Pt* and *Hf-Hf* interactions)

**Figure 2.**
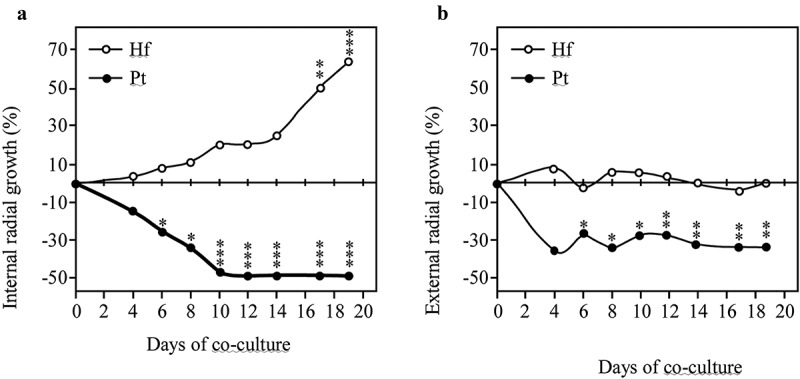
Inner (A) and outer (B) radial growth of *H. fasciculare* (*Hf*) and *P. tinctorius* (*Pt*) in dual culture (*Pt-Hf*), in relation to control colonies *Hf-Hf* and *Pt-Pt*, respectively, during the interaction. Results display mean ± standard error obtained from 12 replicates (two independent experiments). Statistically significant differences of the radial growth in comparison to controls were obtained using one-way ANOVA with Tukey post-test and are shown using * for *p*< 0.05; ** for *p*< 0.01; and *** for *p*< 0.001

Dual cultures between *P. tinctorius – H. fasciculare* (*Pt-Hf* interaction), *P. tinctorius* – *P. tinctorius* (*Pt-Pt* control), and *H. fasciculare* – *H. fasciculare* (*Hf-Hf* control), were established in Petri plates containing MMN. In the presence of *H. fasciculare, P. tinctorius* mycelium showed significant alterations when compared with the *Pt-Pt* control (Figure 1). Although up to 6 d of *Pt-Hf* interaction a radial and uniform *P. tinctorius* growth had been observed, the mycelium in the interacting zone with *H. fasciculare* became denser, more compact and exhibited less air growth when compared to *Pt-Pt* control. These features and a reduction in mycelial extension were particularly notorious after 17 d of *Pt-Hf* co-culture, where a clear distinction between inter-inocula mycelia and diametrically opposed region was detected. When the relative growth of *P. tinctorius* in *Pt-Hf* interaction in relation to *Pt-Pt* control was determined, towards and away the interacting fungi (internal and external radiuses, respectively), a relevant inhibitory effect of *H. fasciculare* on the growth of *P. tinctorius* was obvious (Figure 2). This inhibition occurred prior to mycelial contact between *P. tinctorius* and *H. fasciculare* that only occurred at 22 d of dual-culture, which is typical of an antagonistic interaction at a distance (Boddy 2000). Just after 4 d of dual-culture, the internal radial growth of *P. tinctorius* was inhibited by 14% when compared to *Pt-Pt* control. However, this inhibition only became statistically significant from 6 d of interaction on, reaching the highest internal growth reduction at 12 d (49% of inhibition), the moment after which the hyphal contact between *P. tinctorius – P. tinctorius* colonies did not allow to correctly follow the growth inhibition during interaction. The presence of *H. fasciculare* not only significantly reduced the internal growth of *P. tinctorius* prior to mycelial contact of both fungi, but also the external growth (up to 27%; Figure 2), suggesting the presence of a diffusible or volatile antimicrobial component(s) responsible for the inhibition (Hynes et al. 2007; Uwamori et al. 2015).

*H. fasciculare* cultured with *P. tinctorius* always exhibited a vigorous and increased growth in relation to *Hf-Hf* control (Figure 1). After the physical contact between fungal colonies on *Pt-Hf* co-culture, *H. fasciculare* mycelium kept growing around *P. tinctorius* colony up to the end of 40 d of dual-culture (data not shown) displaying a partial replacement scenario (Boddy 2000; Hiscox et al. 2015). Accordingly, after 17 and 19 d of *Pt-Hf* interaction, *H. fasciculare* has already exhibited more than 50% and 60% of internal radial relative growth, respectively, when compared to *Hf-Hf* controls (Figure 2). In contrast to *P. tinctorius*, no significant differences were found in the external growth of *H. fasciculare* in *Pt-Hf* and *Hf-Hf* dual-cultures (Figure 2).

Interestingly, major differences were detected in the growth of *Pt-Pt* and *Hf-Hf* controls. While an overlap of both *P. tinctorius* mycelia occurred in *Pt-Pt* control just after 10 d of culture, both colonies of *H. fasciculare* never overlapped, appearing to avoid occupying the same space (Figure 1). *H. fasciculare* occupied the entire surface of the culture medium, but the presence of a clear wide line between both colonies remained even after 40 d of culture (results not shown), revealing an avoidance (negative autotropism) mechanism for keeping a suitable distance between growing tips (Glass et al. 2000).

**Figure 3.**
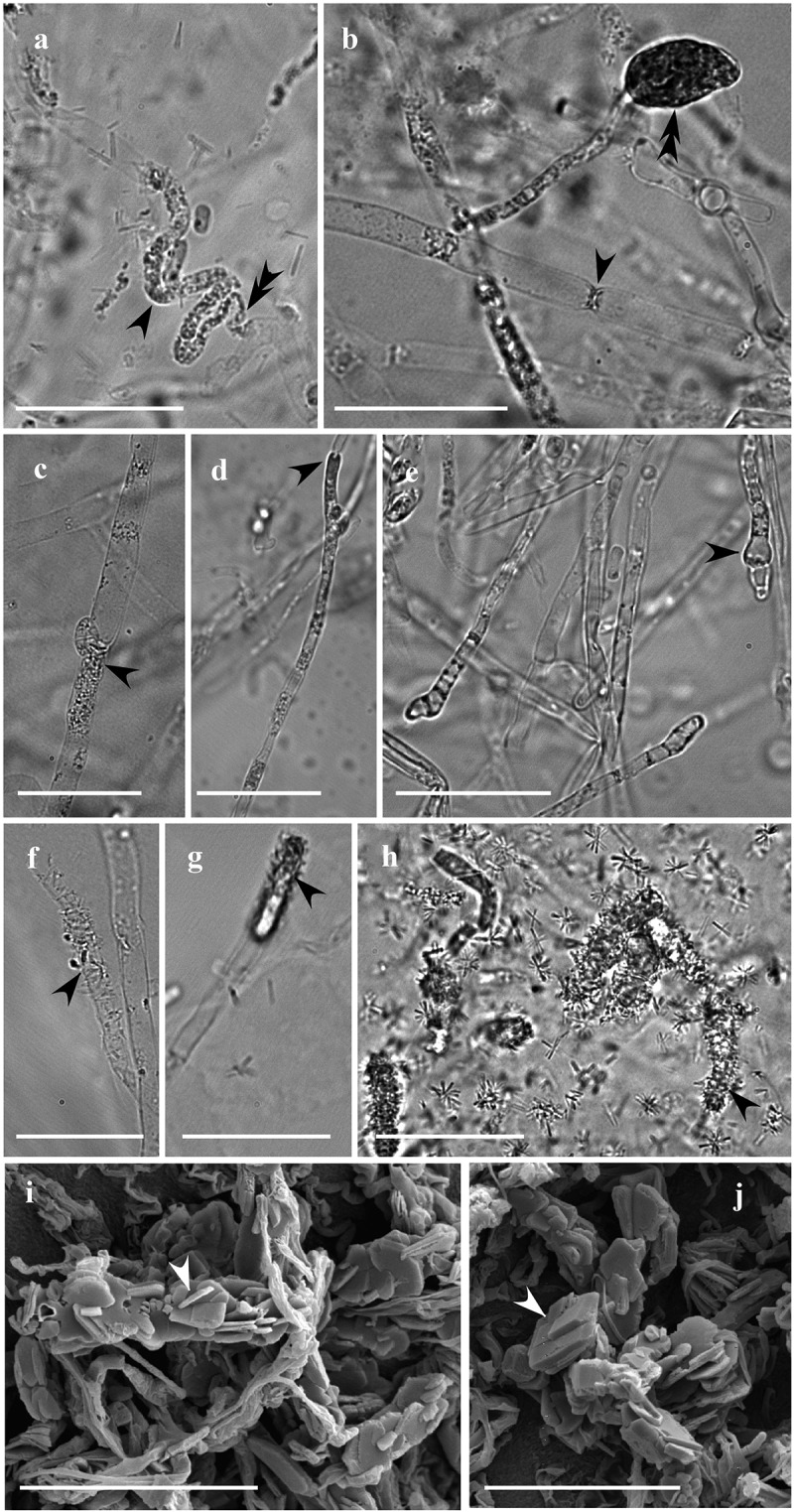
Morphological alterations of *P. tinctorius* and *H. fasciculare* hyphae, observed under light microscopy (A-H) and scanning electron microscopy (I-J), after 25 days of interaction. While *H. fasciculare* hyphae did not change their morphology, *P. tinctorius* hyphae appeared to gain grains (A, C – simple arrows), which are often associated to a pronounced hyphal collapse (A – double arrow), displaying also hyphal constraints (B – simple arrow), partial destruction of the hyphal wall (D – single arrow) and hyphal tips with atypical forms (E – simple arrow) or with vesicle-like structures (B – double arrow). In *Pt-Hf* interaction zone many crystalline structures were adherent to the surface of *P. tinctorius* hyphae (F, G, H – simple arrows). In SEM, these crystals displayed variable dimension (I, J), showing a laminated aspect, and being found free, overlapping or embedded in each other. Bar, 25 µm (A-H) and 20 µm (I, J)

The hyphae morphology in the fungal interacting zones of *Pt-Hf* co-culture, as well as on *Pt-Pt* and *Hf-Hf* controls, was observed after 25 d of interaction, both by optical and scanning electron microscopy (Figure 3). In contrast to the contact zone occurring in *Pt-Pt* and *Hf-Hf* controls, *Pt-Hf* dual-culture exhibited drastic morphological alterations in the interacting hyphae, revealing a hyphal interference antagonism (Boddy 2000). These changes have only occurred in *P. tinctorius* hyphae, which were easily identified by their larger width and presence of clamp connections. The most common alterations were protoplasm granulation, often associated with a pronounced hyphae collapse, and swelling of hyphal apex with the formation of vesicle-like structures (Figure 3A-E). Such alterations have been frequently reported in different incompatibility systems (Inoue et al. 2011; Ujor et al. 2012), including in the ECM *Laccaria laccata* interactions (Werner et al. 2002; Zadworny et al. 2004) and have been related with programmed cell death (PCD) events (Inoue et al. 2011). The presence of many crystals strongly adherent to the surface of *P. tinctorius* interacting hyphae was also observed in *Pt-Hf* interaction (Figure 3F-H), but not in *Pt-Pt* and *Hf-Hf* controls (results not shown). The presence of such crystals in *Pt-Hf* was further confirmed by SEM. The crystals with a laminated aspect displayed variable dimensions and were found free, overlapping or embedded in each other (Figure 3I, J). The X-ray microanalysis by energy dispersive spectroscopy coupled to SEM revealed that these crystals were composed of calcium, being most likely calcium oxalate crystals. No attempt was made to identify the fungus that produces such crystals while in interaction, but the strong adhesion of crystals on the hyphal surfaces of *P. tinctorius* suggests the production by this fungus. However, the question of which fungus is producing it remains. To the best of our knowledge, this is the first report of the production of calcium oxalate crystals following fungus–fungus interaction. However, the production of calcium oxalate has been well documented in saprotrophic, symbiotic (mycorrhizal and lichen) and pathogenic fungi, playing an important role on soil ecology (Dutton and Evans 1996; Michael et al. 2014). Specific environmental conditions and stress factors have been reported to affect oxalate production (Michael et al. 2014). Six ECM and two saprotrophic fungi (in which *H. fasciculare* was included) increased their production of oxalate when exposed to Pb, Cd or As, but ECM species were found to produce more oxalate than saprotrophic ones (Johansson et al. 2008). Accordingly, the likely production of calcium oxalate by the ECM *P. tinctorius* could be a stress response to *H. fasciculare* attack, as described in other ECM stress responses (Johansson et al. 2008), and should be addressed in the future. Other possibility is the production of calcium oxalate crystals due to the artificial conditions (MMN medium) where interactions have occurred. Despite this, the previous pot experiments where both fungi were interacting with each other within a soil environment have indicated that *P. tinctorius* is restrained by *H. fasciculare* presence (Pereira et al. 2011).

Overall, the ability of *H. fasciculare* to antagonise *P. tinctorius* was noticed as an antagonism at a distance followed by partial replacement, where hyphal interference occurs after mycelial contact. Despite the importance of the pairwise interactions in giving important information of the general mechanisms operating between these two fungal species, it doesn’t consider the complexity of the interaction *H. fasciculare-P.tinctorius-C. sativa* that occurs within soil microbial communities. Therefore, extrapolation of the results obtained in this work to field situations cannot be done directly. Further work will be necessary to get insight in the *H. fasciculare*-suppression mechanisms that are operational at the field scale.

## VOCs production during fungal interaction

A group of metabolites that are recognised to be major players of an antagonism at a distance interaction are volatile compounds (Li et al. 2020). However, to date little is known on how inter-specific interactions between saprotrophic and ectomycorrhizal fungi affect volatiles production. In this work, the production of VOCs by *Pt-Hf* interaction and corresponding controls (*Pt-Pt* and *Hf-Hf*) was followed during the time-course of fungal interaction, at 3 (without inhibition), 8 (inhibition with statistical significance), and 14 days (maximal inhibition). The formally and tentatively identified VOCs belong to different chemical classes (Table S1) and the produced VOCs by the three dual-cultures are quali- and semi-quantitatively different (Table 1).

**Table 1.** Volatile profile of *P. tinctorius* and *H. fasciculare* (*Pf-Hf* co-culture) and self-paired interactions (*Pt-Pt* and *Hf-Hf* interactions) at 3, 8 and 14 days of interaction under *in vitro* conditions

Compounds	Area/1000 ± SD								
	Pf-Hf			Hf-Hf			Pt-Pt		
	3 days	8 days	14 days	3 days	8 days	14 days	3 days	8 days	14 days
1-Butanol ^L2^	nd^a^	nd^a^	nd^a^	nd^a^	nd^a^	nd^a^	13.6 ± 7.7^b^	nd^a^	nd^a^
3-Methyl-1-butanol ^L1^	4.23 ± 0.9^a^	293 ± 29^b^	nd^a^	32.6 ± 10.4^a^	nd^a^	nd^a^	nd^a^	33.1 ± 26.2^a^	nd^a^
2-Methyl-1-butanol ^L1^	11.9 ± 2.4^a^	315 ± 60^b^	nd^a^	31.7 ± 2.2^a^	nd^a^	nd^a^	nd^a^	1.75 ± 1.62^a^	nd^a^
1-Octen-3-ol ^L2^	nd^a^	nd^a^	nd^a^	nd^a^	nd^a^	nd^a^	324 ± 245^b^	nd^a^	nd^a^
3-Methylbutanal ^L1^	54.4 ± 5.1^abc^	103 ± 44^bc^	nd^a^	22.9 ± 21.9^a^	120 ± 1^c^	nd^a^	nd^a^	9.20 ± 0.1^b^	nd^a^.
2-Methylbutanal ^L1^	16.7 ± 1.9^a^	139 ± 13 ^c^	nd^a^	91.0 ± 13.8^b^	103 ± 44^bc^	nd^a^	nd^a^	7.97 ± 5.20^a^	nd^a^
Benzaldehyde ^L1^	nd^a^	12.5 ± 3.5^a^	444 ± 49^a^	nd^a^	nd^a^	1801 ± 409^b^	nd^a^	nd^a^	nd^a^
Phenylacetaldehyde ^L1^	nd^a^	nd^a^	66.1 ± 11.4^b^	nd^a^	nd^a^	283 ± 11 ^c^	nd^a^	nd^a^	nd^a^
(*E*)-2-Octenal ^L1^	nd^a^	nd^a^	nd^a^	nd^a^	nd^a^	96.9 ± 40.2^b^	nd^a^	nd^a^	nd^a^
(*E*)-2-Decenal ^L1^	nd^a^	nd^a^	nd^a^	nd^a^	nd^a^	18.3 ± 11.3^b^	nd^a^	nd^a^	nd^a^
3-Methylbutanoic acid ^L2^	nd^a^	nd^a^	nd^a^	nd^a^	nd^a^	nd^a^	nd^a^	nd^a^	51.7 ± 6.0^b^
Benzoic acid ethyl ester ^L2^	nd^a^	nd^a^	nd^a^	nd^a^	nd^a^	103 ± 33^b^	nd^a^	nd^a^	nd^a^
6-Methyl-5-hepten-2-one ^L1^	nd^a^	nd^a^	nd^a^	nd^a^	nd^a^	nd^a^	nd^a^	nd^a^	23.0 ± 1.8^b^
3-Octanone ^L1^	nd^a^	79.3 ± 1.2 ^cd^	nd^a^	56.9 ± 10.6^bc^	42.1 ± 15.6^b^	86.5 ± 0.8^d^	nd^a^	nd^a^	nd^a^
α-Pinene ^L1^	30.1 ± 8.5^ab^	21.2 ± 3.4^ab^	nd^a^	45.1 ± 7.2^b^	19.7 ± 3.6^ab^	nd^a^	42.2 ± 35.5^b^	28.8 ± 3.9^ab^	19.5 ± 6.0^ab^
β-Pinene ^L1^	84.9 ± 4.1^b^	290 ± 2^c^	127 ± 29^b^	270 ± 26 ^c^	355 ± 45^d^	80.1 ± 5.3^b^	nd^a^	nd^a^	nd^a^
Limonene ^L1^	125 ± 7^a^	243 ± 12^b^	119 ± 7^a^	126 ± 12^a^	255 ± 31^b^	234 ± 35^b^	156 ± 49^a^	127 ± 17^a^	159 ± 2^a^
Linalool ^L1^	55.6 ± 32.2^b^	176 ± 17 ^c^	nd^a^	65.3 ± 8.5^b^	231 ± 22^d^	nd^a^	nd^a^	nd^a^	nd^a^
Menthol ^L1^	nd^a^	nd^a^	nd^a^	nd^a^	nd^a^	nd^a^	nd^a^	nd^a^	21.3 ± 3.0^b^
Clovene ^L1^	nd^a^	47.9 ± 16.4^d^	11.1 ± 1.2^ab^	28.0 ± 2.8^bcd^	17.3 ± 11.3^abc^	33.2 ± 1.7 ^cd^	nd^a^	nd^a^	nd^a^
β-Elemene ^L2^	nd^a^	18.9 ± 1.1^c^	24.5 ± 0.4^d^	nd^a^	16.4 ± 0.8^b^	nd^a^	nd^a^	nd^a^	nd^a^
Copaene ^L2^	29.7 ± 15.7^bc^	22.3 ± 2.1^ab^	nd^a^	nd^a^	20.6 ± 2.1^ab^	nd^a^	51.1 ± 25.9^c^	11.3 ± 1.7^ab^	nd^a^
Longifolene ^L2^	45.0 ± 19.2 ^cd^	29.8 ± 1.1^abc^	12.5 ± 1.8^a^	32.7 ± 6.6^bc^	23.1 ± 1.1^ab^	16.0 ± 1.1^ab^	53.2 ± 12.2^d^	21.5 ± 3.2^ab^	12.0 ± 0.8^a^
β-Caryophyllene ^L1^	55.6 ± 8.6^bc^	105 ± 8^c^	103 ± 0.4^c^	93.4 ± 10.9^c^	64.9 ± 45.7^c^	12.6 ± 0.1^ab^	nd^a^	nd^a^	nd^a^
Aromadendrene ^L2^	nd^a^	11.2 ± 4.0^a^	127 ± 53^b^	18.2 ± 1.4^a^	9.50 ± 1.2^a^	42.6 ± 4.3^a^	nd^a^	nd^a^	nd^a^
Valencene ^L1^	5.82 ± 1.82^ab^	89.8 ± 9.0^e^	39.4 ± 4.9 ^cd^	38.4 ± 5.8^bcd^	52.3 ± 27.9^d^	16.9 ± 0.9^abc^	nd^a^	nd^a^	nd^a^
β-Farnesene ^L2^	nd^a^	307 ± 14 ^c^	nd^a^	nd^a^	128 ± 9^b^	nd^a^	nd^a^	nd^a^	nd^a^
α-Muurolene ^L1^	nd^a^	2517 ± 454 ^c^	2593 ± 332 ^c^	11.2 ± 1.8^a^	1788 ± 217^b^	496 ± 74^a^	nd^a^	nd^a^	nd^a^
δ-Cadinene ^L2^	nd^a^	32.6 ± 4.3^d^	32.6 ± 4.7^d^	nd^a^	22.7 ± 2.6 ^c^	14.0 ± 0.7^b^	nd^a^	nd^a^	nd^a^
Unidentified nitrogen-like compound 1 ^L4^	255 ± 31^a^	3234 ± 1692^a^	33,320 ± 283 ^c^	464 ± 85^a^	1841 ± 518^a^	13,030 ± 5233^b^	nd^a^	nd^a^	nd^a^
1,4-Dichlorobenzene ^L2^	nd^a^	nd^a^	770 ± 97^b^	nd^a^	nd^a^	871 ± 86^b^	nd^a^	nd^a^	2268 ± 403 ^c^
2,6-Dichloroanisole ^L2^	nd^a^	nd^a^	41.2 ± 0.0^b^	nd^a^	nd^a^	476 ± 37 ^c^	nd^a^	nd^a^	nd^a^
2,6-Dichlorobenzaldehyde ^L2^	nd^a^	nd^a^	183 ± 46 ^c^	nd^a^	nd^a^	127 ± 8^b^	nd^a^	nd^a^	nd^a^
3-Chloro-4-metoxybenzaldehyde ^L1^	nd^a^	nd^a^	921 ± 69 ^c^	nd^a^	nd^a^	360 ± 16^b^	nd^a^	nd^a^	nd^a^
Unidentified Chlorinated-like compound 2 ^L4^	nd^a^	52.9 ± 13.8^a^	315 ± 109^b^	nd^a^	650 ± 151 ^c^	186 ± 26^ab^	nd^a^	nd^a^	nd^a^
2,4-Dichloro-3-methoxy benzoic acid ethyl ester ^L2^	nd^a^	nd^a^	9.50 ± 1.58^b^	nd^a^	nd^a^	55.5 ± 6.6 ^c^	nd^a^	nd^a^	nd^a^
Toluene ^L1^	50.9 ± 0.1^a^	79.1 ± 8.1^ab^	nd^a^	69.7 ± 7.1^ab^	54.0 ± 4.7^a^	nd^a^	47.1 ± 21.6^a^	182 ± 121^b^	nd^a^
Ethylbenzene ^L1^	41.6 ± 14.8^b^	34.2^b^± 4.8	nd^a^	33.2 ± 16.4^b^	32.2 ± 6.1^b^	nd^a^	nd^a^	54.1 ± 8.7^b^	nd^a^
ρ-Xylene ^L1^	572 ± 207^bc^	168 ± 17^ab^	nd^a^	422 ± 172^abc^	125 ± 2^a^	nd^a^	772 ± 248 ^c^	217 ± 25^ab^	89.2 ± 19.9^a^
σ-Xylene ^L1^	75.8 ± 23.0^b^	nd^a^	nd^a^	72.0 ± 28.5^b^	nd^a^	nd^a^	150 ± 41 ^c^	nd^a^	nd^a^
Naphthalene ^L1^	599 ± 229^a^	1646 ± 11^b^	586 ± 62^a^	261 ± 4^a^	1240 ± 132^b^	679 ± 27^a^	600 ± 262^a^	1601 ± 34^b^	632 ± 48^a^

Area expressed as arbitrary units; SD = standard deviation of three determinations; nd = not detected; values not sharing the same superscript letters (a–f) within the horizontal line are different according to the Tukey test (p < 0.05). L1: Identified metabolites (GC-MS analysis of the metabolite of interest and a chemical reference standard of suspected structural equivalence, with all analyses performed under identical analytical conditions within the same laboratory) (Viant et al. 2017); L2: Putatively annotated compounds (spectral (MS) similarity with NIST database), when standards were not commercially available (Viant et al. 2017); L4: Unidentified (Viant et al. 2017).

During *Hf-Hf* interaction, only (*E*)-2-octenal, (*E*)-2-decenal and benzoic acid ethyl ester were exclusively detected; while 1-butanol, 1-octen-3-ol, 3-methyl butanoic acid, 6-methyl-5-hepten-2-one and menthol were solely observed in *Pt-Pt* interaction (Table 1). No specific compound was detected during *Pt-Hf* interaction. In contrast, Hynes et al. (2007) reported the exclusive production of 10 VOCs after interaction of *H. fasciculare* with other wood decay fungus, *Resinicium bicolour*, when compared with single-species controls. The unidentified nitrogen-like compound 1 was found to be the most abundant VOC produced by *H. fasciculare* (54% of the total volatile fraction in *Hf-Hf* interaction), followed by α-muurolene and naphthalene; while *P. tinctorius* produced the highest amounts of naphthalene and 1,4-dichlorobenzene. Results also showed that *H. fasciculare* produces a rich variety of sesquiterpenes, aldehydes and chlorine compounds, whereas the ECM *P. tinctorius* produces a higher diversity of alcohols, less terpenes, fewer aldehydes and sesquiterpenes. The production of a high number of sesquiterpenes by *H. fasciculare* has already been described (Hynes et al. 2007) and is in accordance with the report that saprophytes release higher amounts of sesquiterpenes than ECM (Müller et al. 2013). The role of sesquiterpenes as antimicrobial compounds has been reviewed by Kramer and Abraham (2012). Most of the VOCs produced by *H. fasciculare* were already recognised as having antimicrobial activity, namely limonene (Ünal et al. 2012), linalool (Bagamboula et al. 2004), longifolene (Tsuruta et al. 2011), β-caryophyllene (Minerdi et al. 2009), and cadinene (Troncoso et al. 2011). Among these, β-caryophyllene is particularly suited as a belowground signal due to its diffusion properties (Hiltpold and Turlings 2008). The reduced number of sesquiterpenes detected in *P. tinctorius* is quite surprising, due to their reported role in below mycorrhizal signalling and lateral root stimulation (Ditengou et al. 2015). The production of VOCs also changed along fungal interaction (Table 1; Fig. S1). In *Pt-Hf* interaction the number of VOCs, mainly sesquiterpenes, increased from 18 (at 3 d) to 26 (at 8 d) and declined afterwards to 20 (at 14 d). In contrast, the number of VOCs was found to slightly change during *Hf-Hf* and *Pt-Pt* interactions (Fig. S1).

**Figure 4.**
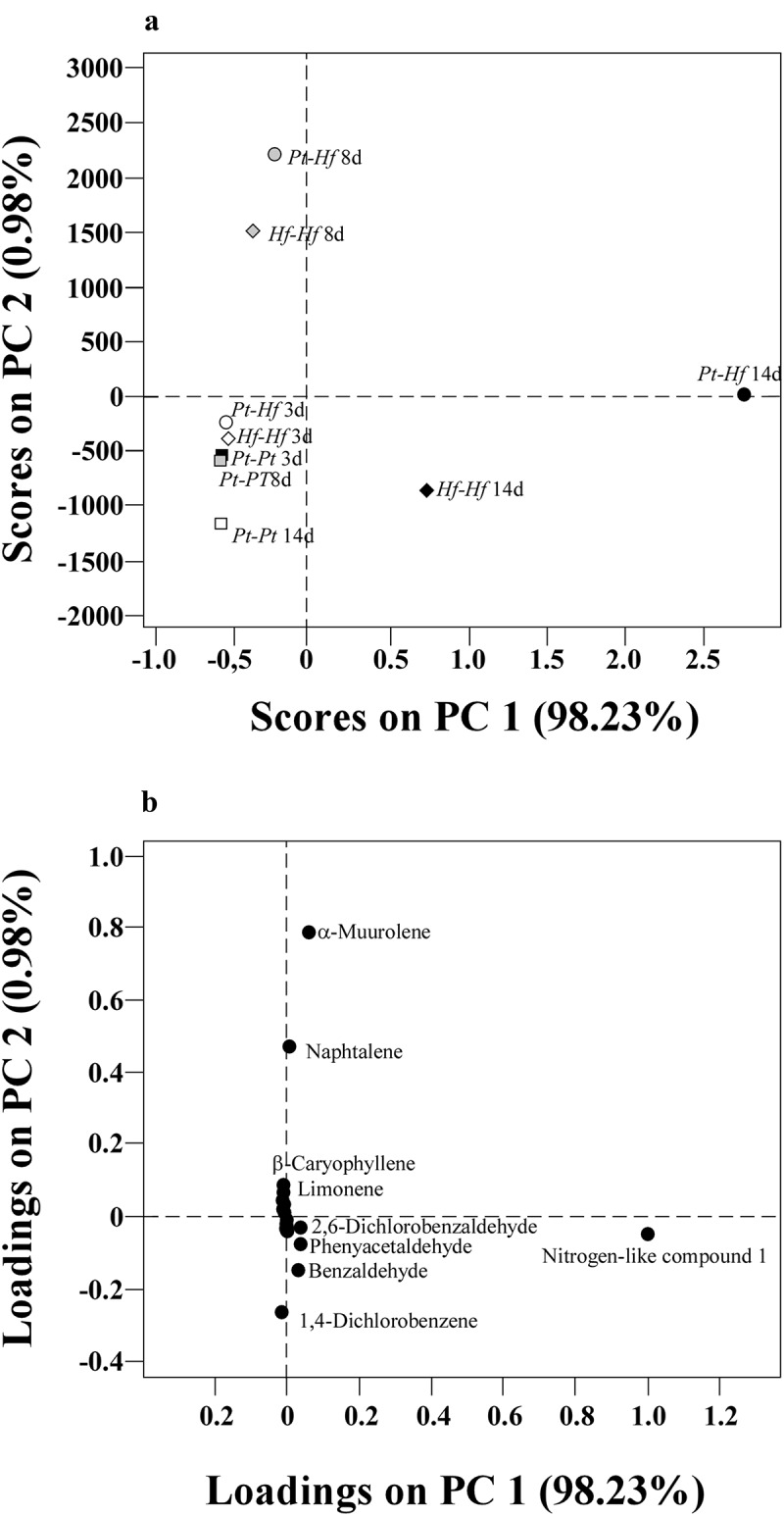
PCA scores (A) and loadings (B) plots obtained from the mean-centred area-arbitrary units of the quantified volatile compounds obtained in the co-cultures of *P. tinctorius* – *H. fasciculare* (Pt-Hf), *P. tinctorius* – *P. tinctorius* (Pt-Pt), and *H. fasciculare* – *H. fasciculare* (Hf-Hf), after 3-, 8- and 14 days of interaction under *in vitro* conditions. The PCA factors explain 99.21% of the total variance. The PCA was performed taking into account 33 volatile compounds of the Table S1. Volatiles 3-methylbutanoic acid, 6-methyl-5-hepten-2-one, menthol, longifolene, 1,4-dichlorobenzene, toluene, ethylbenzene and σ-xylene, were excluded from this target analysis

For understanding the dynamics of VOCs production over time and the potential role of antagonising compounds, the changing trend of each individual VOCs was evaluated during fungal interactions. In this study, any compound was *de novo* produced in *Pt-Hf* interaction as compared to controls *Hf-Hf* and *Pt-Pt* interactions; but VOCs production considerably changed during different interaction stages. The PCA analysis performed from the pre-processed raw data (untargeted analysis) allowed the discrimination of volatile profiles after 8 and 14 days in *Pt-Hf* and *Hf-Hf* interactions, while all volatile profiles derived after 3 days of co-cultures and from all *Pt-Pt* interaction periods remained undifferentiated (Fig. S2). A PCA performed using 41 variables (peak areas of 41 compounds) show a higher variance, 78.07%, considering the two principal components (PC1 and PC2) (Table S1). The variables 3-methylbutanoic acid, 6-methyl-5-hepten-2-one, menthol, longifolene, 1,4-dichlorobenzene, toluene, ethylbenzene and σ-xylene were not included in subsequent target analysis, as they presented a communality value lower than 0.56. The representation of variables and fungal interactions during 3, 8 and 14 days, using the first two PCs of targeted analysis, corroborated the clear discrimination of *Pt-Hf* and *Hf-Hf* interactions along time (Figure 4). In general, both PCA approaches resulted in similar scores distribution pattern, certifying the strong relation between *Pt-Hf* and *Hf-Hf* after 8 and 14 days of interaction. The first principal component (PC1), that explains 98.23% of total variance, is positively related with unidentified nitrogen-like compound 1 and fungal cultures with 14 days of interaction. The second principal component (PC2), that explains 0.98% of total variance, is positively correlated with α-muurolene and naphthalene, and cultures with 8 days of interaction. *Pt-Pt* interactions at 3, 8 and 14 days are placed closer in the projection, negatively related to PC1 and PC2.

Most volatiles that were induced during interaction between *Pt-Hf* seem to be produced by *H. fasciculare*, since they were never been detected in *P. tinctorius* controls. In contrast, some VOCs produced by *H. fasciculare* do not appear to be produced during *Pt-Hf* interaction, namely (*E*)-2-octenal, (*E*)-2-decenal and benzoic acid ethyl ester (Table 1). Changes in the volatile profile in each stage of *Pt-Hf* interaction were coincident with *P. tinctorius* growth inhibition (3 d with no inhibition, when compared to 8 d corresponding to significant inhibition, and 14 d to maximal inhibition). Although induced VOCs could have been already produced by *H. fasciculare* normal metabolism, their increased production in the presence of *P. tinctorius* may play a role in the antagonism process, during the different interaction stages. Altogether, the results suggest that the production of VOCs is highly dependent on the fungal interaction dynamics, as already been pointed by several studies (Hynes et al. 2007; Evans et al. 2008). From all the detected VOCs, α-muurolene and unidentified nitrogen-like compound 1, seem to be the most discriminant compounds that could play a role in the antagonism process of *H. fasciculare* against *P. tinctorius* at 8 d a 14 d, respectively. Accordingly, many sesquiterpenes (including α-muurolene) have been reported to have antimicrobial or insecticidal effects, playing a key role in the communication between fungi, insects and plants (reviewed by Kramer and Abraham 2012). For example, α-muurolene was reported to be produced when the mycelia of *H. fasciculare* and *Resinicium bicolour* interacted but was not formed by *R. bicolour* (Hynes et al. 2007). Similarly, nitrogen-containing VOCs have been reported to exhibit antimicrobial activity against several fungal phytopathogens (Chen et al. 2008; Haidar et al. 2016). However, more important than the specific contribution of each volatile for the inhibitory activity, the total mixture of compounds would define the antagonistic activity of the final blend. *H. fasciculare* emits a large battery of antimicrobial compounds that acting together can result in a higher antagonistic potential, when compared to *P. tinctorius* that is a weak VOCs emitter. Apart from VOCs, other secondary metabolites might also have contributed to the inhibitory activity displayed by *H. fasciculare* against *P. tinctorius*. Indeed, there are some reports indicating the capacity of *H. fasciculare* to produce a diverse array of antimicrobial compounds from several chemical classes including benzoic acids, such as 3,5 dichloro-4-methoxy benzoic acid, and sesquiterpenoids (Aqueveque et al. 2006; SAA et al. 2020). Furthermore, *H. fasciculare* often produce extracellular enzymes to attack competitor mycelium (Hiscox and Boddy 2017), and thus, their involvement in the inhibition of *P. tinctorius* cannot be completely excluded as well.

## Conclusions

The growth of *P. tinctorius* is significantly reduced by *H. fasciculare* after six days of co-inoculation and become even more significant through time. *P. tinctorius* also formed vesicles and calcium oxalate crystals in the presence of *H. fasciculare*, which are described as mechanisms of stress adaption by fungi. The emission of volatile compounds changed over the interaction *P. tinctorius* – *H. fasciculare*, being *P. tinctorius* growth inhibition coincident with an increase in nitrogen-containing compounds and sesquiterpenes class compounds produced by *H. fasciculare*. These compounds, which are recognised as having antimicrobial activity, might play an important role on *P. tinctorius* growth inhibition.

## Supplementary Material

Supplemental Material

## Appendix A. Supplementary data

Supplementary material related to this article can be found, in the online version.
