# Supplementary material for: In vitro interactions between the ectomycorrhizal Pisolithus tinctorius and the saprotroph Hypholoma fasciculare fungi: morphological aspects and volatile production
Source: Mycology. 2021 Feb 27;12(3):216–29. doi: 10.1080/21501203.2021.1876778 (PMC8451600; doi:10.1080/21501203.2021.1876778)
Supplement: Supplemental Material [file TMYC_A_1876778_SM4562.docx]

**Table S1 -** Volatile compounds detected in this study: retention time (RT), kovats retention indices (RI), selected ions used as *m/z* identifiers and quantifiers (QI) and identification method (ID).

| **RT (min)** | **RI calc^A^** | **RI lit^B^** | **Compounds** | **QI (*m/z*)** | **ID^C^** |
| --- | --- | --- | --- | --- | --- |
|  |  |  | **Alcohols** |  |  |
| 2.59 | - | 659 | 1-Butanol ^L2^ | 41/43/56 | MS (73.7/83.3) |
| 3.52 | - | 736 | 3-Methyl-1-butanol ^L1^ | 42/43/55/70 | STD/MS |
| 3.57 | - | 739 | 2-Methyl-1-butanol ^L1^ | 56/57/70 | STD/MS |
| 9.39 | 999 | 980 | 1-Octen-3-ol ^L2^ | 43/57/72 | MS (78.2/82.7) |
|  |  |  | **Aldehydes** |  |  |
| 2.48 | - | 652 | 3-Methylbutanal ^L1^ | 43/43/44/58 | STD/MS |
| 2.57 | - | 662 | 2-Methylbutanal ^L1^ | 41/57/58 | STD/MS |
| 8.45 | 966 | 962 | Benzaldehyde ^L1^ | 77/105/106 | STD/MS |
| 10.84 | 1048 | 1045 | Phenyl acetaldehyde ^L1^ | 91/92/120 | STD/MS |
| 11.26 | 1062 | 1062 | (*E*)-2-Octenal ^L1^ | 41/55/70/83 | STD/MS |
| 17.12 | 1266 | 1263 | (*E*)-2-Decenal ^L1^ | 41/43/55/70/83 | STD/MS |
|  |  |  | **Acid** |  |  |
| 5.79 | 866 | 863 | 3-Methylbutanoic acid ^L2^ | 41/43/60 | MS (87.7/88.1) |
|  |  |  | **Esters** |  |  |
| 14.52 | 1174 | 1171 | Benzoic acid ethyl ester ^L2^ | 77/105/122 | MS (85.4/88.4) |
|  |  |  | **Ketones** |  |  |
| 9.06 | 987 | 986 | 6-Methyl-5-hepten-2-one ^L1^ | 41/43/55/69/108 | STD/MS |
| 9.49 | 1002 | 986 | 3-Octanone ^L1^ | 43/57/71/72/99 | STD/MS |
|  |  |  | **Terpenes** |  |  |
| 7.94 | 948 | 937 | α-Pinene ^L1^ | 91/92/93 | STD/MS |
| 9.55 | 1004 | 979 | β-Pinene ^L1^ | 41/69/93 | STD/MS |
| 10.78 | 1046 | 1030 | Limonene ^L1^ | 68/93 | STD/MS |
| 12.93 | 1119 | 1099 | Linalool ^L1^ | 55/71/93/121 | STD/MS |
| 14.75 | 1182 | 1169 | Menthol ^L1^ | 71/81/95 | STD/MS |
|  |  |  | **Sesquiterpenes** |  |  |
| 20.39 | 1389 | 1425 | Clovene ^L1^ | 161/189/204 | STD/MS |
| 20.49 | 1393 | 1391 | β-Elemene ^L2^ | 41/68/81/93 | MS (85.1/86.6) |
| 20.66 | 1399 | 1376 | Copaene ^L2^ | 105/119/161 | MS (81.6/85.0) |
| 21.30 | 1425 | 1419 | β-Caryophyllene ^L1^ | 41/91/93/133 | STD/MS |
| 21.60 | 1437 | 1405 | Longifolene ^L2^ | 91/93/94/161 | MS (85.2/87.4) |
| 21.79 | 1445 | 1440 | Aromadendrene ^L2^ | 41/91/161 | MS (86.1/88.8) |
| 21.89 | 1449 | 1492 | Valencene ^L1^ | 105/161/204 | STD/MS |
| 22.51 | 1474 | 1457 | β-Farnesene ^L2^ | 41/69/93 | MS (86.2/88.3) |
| 23.26 | 1504 | 1499 | α-Muurolene ^L1^ | 105/161 | STD/MS |
| 24.20 | 1544 | 1524 | δ-Cadinene ^L2^ | 105/119/134/161 | MS (80.1/84.7) |
|  |  |  | **Nitrogen compounds** |  |  |
| 22.42 | 1470 | - | Unidentified 1 ^L4^ | 203 | - |
|  |  |  | **Chlorinated compounds** |  |  |
| 9.97 | 1019 | 1021 | 1,4-Dichlorobenzene ^L2^ | 111/146/148 | MS (92.2/93.3) |
| 15.27 | 1199 | 1185 | 2,6-Dichloroanisole ^L2^ | 133/161/176 | MS (89.6/92.6) |
| 17.00 | 1262 | - | 2,6-Dichlorobenzaldehyde ^L2^ | 173/174/175 | MS (88.8/90.7) |
| 22.29 | 1465 | - | 3-Chloro-4-methoxybenzaldehyde ^L1^ | 169/170/171 | STD/MS |
| 25.87 | 1616 | - | Unidentified 2 ^L4^ | 206 | - |
| 26.55 | 1646 | - | 2,4-Dichloro-3-methoxy benzoic acid ethyl ester^L2^ | 203/205/248 | MS (81.5/81.8) |
|  |  |  | **Other compounds** |  |  |
| 3.97 | - | 763 | Toluene ^L1^ | 91/92 | MS (87.5/91.6) |
| 5.99 | 874 | 855 | Ethylbenzene ^L1^ | 91/106 | MS (87.6/93.1) |
| 6.23 | 885 | 865 | ρ-Xylene ^L1^ | 91/106 | MS (92.8/93.4) |
| 6.83 | 908 | 887 | σ-Xylene ^L1^ | 91/106 | MS (89.1/90.7) |
| 15.51 | 1208 | 1182 | Naphthalene ^L1^ | 127/128/129 | MS (92.3/93.7) |

**^A^**RIcalc: retention indices calculated from C8 to C20 n-linear alkanes with VF-5 ms capillary column.

^B^RIlit: retention indices reported in the literature for VF-5 ms capillary column or equivalent. **^C^**ID = Identification method. Compounds were identified by comparing (1) their retention times with those of authentic compounds (STD), (2) the retention indices with those from literature data, and (3) the MS fragmentation pattern with those of STD and mass spectra database performed using NIST 14 spectral database, considering fit and retrofit values >70 %. ^L1^: Identified metabolites (GC-MS analysis of the metabolite of interest and a chemical reference standard of suspected structural equivalence, with all analyses performed under identical analytical conditions within the same laboratory) (Viant *et al*. 2017); ^L2^: Putatively annotated compounds (spectral (MS) similarity with NIST database), when standards were not commercially available (Viant *et al*. 2017); ^L4^: Unidentified (Viant *et al*. 2017).

**Figure S1** - Venn diagrams indicating the number of volatile compounds specific or shared among the different co-cultures [*P. tinctorius* – *H. fasciculare* (*Pt-Hf*), *P. tinctorius* - *P. tinctorius* (*Pt-Pt*), and *H. fasciculare* - *H. fasciculare* (*Hf-Hf*)] at 3, 8 and 14 days of interaction under *in vitro* conditions. Bold numbers indicate the total number of volatile compounds identified for each co-culture.


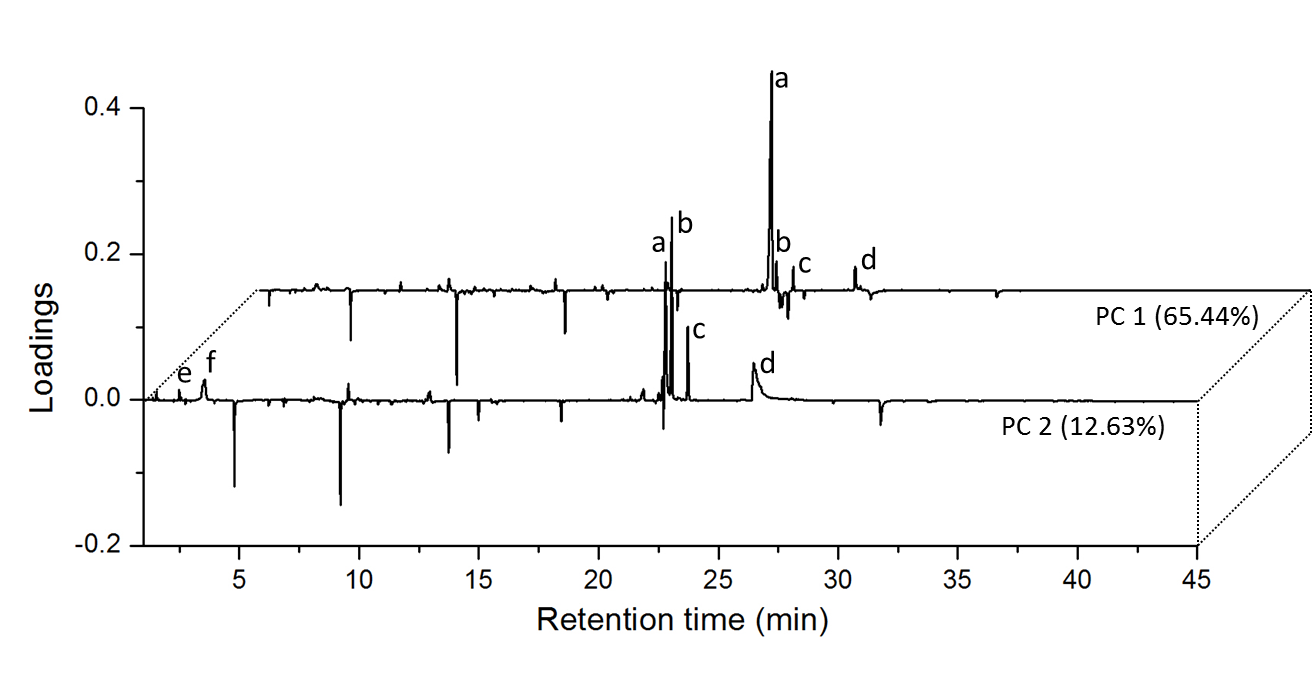

*Pt-Hf* 3d

*Pt-Hf* 8d

*Pt-Hf* 14d

*Hf-Hf* 14d

*Hf-Hf* 3d

*Hf-Hf* 8d

*Pt-Pt* 14d

*Pt-Pt* 3d

*Pt-Pt* 8d

Medium 14d

Medium 3d

Medium 8d

**A**

**B**

**Figure S2 -** PCA scores (A) and loadings (B) plots obtained from all aligned, intensity-normalized and mean-centered chromatograms obtained in the co-cultures of *P. tinctorius* – *H. fasciculare* (*Pt-Hf*), *P. tinctorius* - *P. tinctorius* (*Pt-Pt*), and *H. fasciculare* - *H. fasciculare* (*Hf-Hf*), after 3-, 8- and 14 days of interaction under *in vitro* conditions. The PCA factors explain 78.07% of the total variance. The most relevant compounds able to contribute to sample differentiation are a) nitrogen-like compound 1 (*m/z* 203); b) β-caryophyllene; c) α-muurolene; d) chloric-like compound 1 (*m/z* 206); e) 3-methylbutanal and f) 2-methyl-1-butanol. Other relevant compounds were recognized as chemical noise.

**References**

Viant MR, Kurland IJ, Jones MR, Dunn WB 2017. How close are we to complete annotation of metabolomes? Curr Opin Chem Biol 36: 64–69
